# An Epitome of Surgery

**Published:** 1861-01

**Authors:** 


ART. VI.—*An Epitome of Surgery.* By J. BEADNELL GILL, M.D. 36mo., pp. 94. H. Baillière, 1860.

WE learn from the title-page of this book that this is not the only attempt on the part of Dr. Beadnell Gill at authorship. His first publication was an "Epitome of Botany;" and it would also appear that he had been "Late House Surgeon, etc. etc. at the London Hospital." What the "etc. etc." mean we are not permitted to know; and as we do not care about conjectures where facts alone can be of use, we shall not stop to inquire. Perhaps Dr. Beadnell Gill may condescend to enlighten us upon the subject in some future edition of his work. We regret the omission, simply because we are anxious to place before our readers the full claims of the author to their respect and confidence as a writer upon so important a subject. Mr. Baillière deserves great praise for bringing out the work simultaneously at London, Paris, Madrid, and New York, thus laying it literally at the feet of every physician panting for knowledge, both in the Old World and in the New. But, above all, has he entitled himself to the gratitude of the profession for his ready appreciation of the merits of this work, and for the handsome manner in which, to use a somewhat hackneyed phrase, it has been gotten up.

Our readers have often been told that there is no royal road to knowledge, but that its paths are devious and rugged, if not actually strewn with thorns and briers. They will therefore be not a little gratified to learn that there is now a macadamized road to at least one branch of science, namely, that of surgery, an exploit which a benificent Providence had reserved for the present age, its happy instrument being Dr. Beadnell Gill, the author of the work under consideration. In a preface, remarkable for its brevity, dated "Dover, September, 1860," he modestly remarks:—

> "If I have, in any degree, macadamized the road to the theory of surgery, I have accomplished my wishes. I must, however, remind the student that a practical acquaintance with the science is only to be obtained in the wards, the dissecting and out-patients' rooms, and the dead-house."

Dr. Beadnell Gill should have no cruel apprehensions about the success of his work. We trust that the medical press of this and other countries may speedily relieve his mind of any suspense or misgiving upon the subject. The work is a master-piece of its kind, putting to shame and confusion all its predecessors in the same branch of science; and our only wonder is that, when he had finished his "macadamized road," he did not, like Theophrastus Bombastus Paracelsus, kindle a big fire and throw into it the now obsolete and speedily-to-be-forgotten tomes of Boyer,

Nélaton, Velpeau, Langenbeck, Chelius, Miller, Ferguson, Gross, Erichsen, and similar dreamers and impostors, who have done so much to defraud the student of his time and money. Within the compass of ninety-six 36mo. pages, of which two are occupied with the index, Dr. Beadnell Gill has compressed all that is really necessary to be known of the art and science of surgery, and that, too, for the very reasonable sum of **One Shilling** of her Britannic Majesty's money. Surely the whole profession should rise up as one man and call the author blessed. We trust that Dr. Beadnell Gill has retained the right of translation and reprint of his work, now so common among his brethren; otherwise it can hardly fail to become an object of piracy with the various nations of the earth, in their desire to possess so great a treasure. Mr. Baillière will no doubt see that his protegé is substantially rewarded for his exhausting labors and anxiety. If the author lived in this country, we should advise him to take out a patent right for his cerebral condenser, by whose wonderful agency he has succeeded in supplying the world with this "macadamized road to the theory of surgery."

Knowing that the work of the immortal surgeon of Dover will speedily find its way into the hands of our readers, it would be idle to indulge in extracts. We cannot, however, forbear giving the following passages as specimens of his wonderful compressing faculties:—

"INGROWING OF NAILS:—*Treatment.* Insert a small piece of lint soaked in liq. arsenical, as far as possible between nail and rising granulations. Cures in about a fortnight. Extraction of a nail or even part of it is barbarous and unnecessary.

"ONYCHIA:—Maligna. Unhealthy ulcers about roots of nails—*Treatment.*—Liq. arsen. locally.

"WHITLOW:—Abscess about fingers. Three kinds: Cutaneous, Subcutaneous, and Thecal:—*Treatment.*—First two, poultice and shave off cuticle; third, see page 33."

In taking leave of our author we trust that we shall soon have the pleasure of greeting him again; for it cannot be supposed that he will relax his efforts to benefit his profession. A man who is so adroit in macadamizing would indeed be reckless alike to his duty and to his interest if he did not try his hand in simplifying some of the other branches of medical science. A macadamized road to the theory of medicine, obstetrics, physiology, or chemistry could not fail to confer upon the name of Beadnell Gill additional lustre. Our only fear is that he may overtax his brain, and thus become an early martyr to the cause of science.

To his present performance the distinguished author may proudly apply the lines of the poet:—

"Jamque opus exegi; quod nec Jovis ira, nec ignis,
Nec poterit ferrum, nec edax abolere vetustas."

